# Toothache: Its Diagnosis and Treatment

**Published:** 1892-04

**Authors:** A. V. Elliott

**Affiliations:** Florence, Italy


### TOOTHACHE: ITS DIAGNOSIS AND TREATMENT.[1]

BY DR. A. V. ELLIOTT, FLORENCE, ITALY.

Mr. President,—What I had in view when I gave the title of the paper I intended to read before this Society to my friend, Dr. Bryan, the secretary, was the exigencies or emergencies in dental practice, more particularly in regard to what is generally understood as toothache: how quickest to diagnosticate and give relief. But I find the subject almost too comprehensive to be classed within such limits, and so I fear I must trespass upon your indulgence a little longer than I had intended. As human beings of the nineteenth century, and particularly as dentists, we are, of course, familiar with the subject. It is one, I think, of great importance to us, and especially to those of us who practise in such holiday centres as Lucerne, where so many go for health and pleasure; and of the many, there must naturally be a certain percentage of unfortunates whose anticipated pleasure has been turned to pain and misery by the toothache. But happily, in the providential arrangement of circumstances, it is within the province of the modern dentist to give relief to a greater or less extent. It therefore behooves us for the sake of humanity to lift the load, so to speak, from the weary sufferer as expeditiously as possible.

It is because I want to do my whole duty in this respect—to humanity and myself—that I have chosen this subject, so that I might give you the benefit of my experience for what it is worth, but more particularly so that I might receive in exchange from you the methods and remedies found most effective in your experience.

Toothache proper means inflammation either of the pulp or alveolo-dental membrane. Inflammation, as we all know, is that condition of living tissue which includes pain, heat, tension, etc.,—a want of physiological harmony in the parts,—and is occasioned by anything which would produce that want of harmony or health, as the intrusion of foreign bodies,—thermal shock, septic poison, etc.

The first and most important thing to consider when a case is brought before our notice is the diagnosis. To the patient who comes for relief, no matter what the cause of the pain, it is to him simply a toothache and nothing more, and that is sufficient. But to us as diagnosticians it is a matter of importance to know for a

---

[1] Read before the American Dental Society of Europe, at Heidelberg, August 5, 1891.

certainty what the real cause of the pain is: whether, for example, it arises from irritation of the pulp of the tooth from exposure, or of the periodental membrane, or because of some obscure pathological condition elsewhere. The diagnosis is not always an easy matter. We sometimes have to grope our way very cautiously to arrive at a full assurance of being on the right track. We must isolate the seat of trouble by elimination or exclusion. Ordinarily, when the patient presents for relief, we have little difficulty in discovering what the matter is. We find the cavity, and within it a pulp in a state of irritation; the proper remedy is applied, and the patient goes gratefully away. But we have mouths presented to us sometimes which embarrass us. There are crowns in every state of disintegration; root-canals in all stages of putrescence and the development of gaseous products. The patient suffers, and we are expected to give relief. We must get at the seat of this particular trouble by elimination, and leave the other potentialities for future development.

Again, there are more cases in ordinarily good mouths having no open cavities, but many teeth filled. There is at first sight no appearance of anything wrong, yet the patient suffers. The pain may come from exposure or near exposure of the pulp through a prolongation of a cavity not apparent to the eye, or the pain may arise from a metal filling placed too close to the pulp without adequate protection, or it may be developed from incipient inflammation about the root of a dead tooth. Impacted wisdom teeth are frequently painful to the patient, and osseous deposits in the pulp-chamber and canals; or the pain might have its origin somewhere else besides the teeth, and is felt in a tooth or teeth through the reflex action of the nerves. Rheumatic, gouty, hysterical, and neuralgic people, and women during gestation, are subjects of these irregular phenomena. I might include among the possibilities of pain from an obscure source fillings of different metals in contact, or near by, producing an electro-chemical action. This, I think, is more theoretical than real. I have never personally seen such a case.

In obscure cases we can be helped at times by questioning the patient as to the history of the trouble.

In reducing this matter of diagnosis and treatment to general principles, let us commence with the simplest first. Fortunately for us, these cases of difficult diagnosis are rare. The patients who come to us for relief are usually suffering from either inflammation of the pulp or of the alveolo-dental membrane. My plan is to remove the *débris* and decalcified dentine in the cavity, being careful not to wound the pulp, and then fill with oxyphosphate, leaving enough of the decalcified dentine over the nerve to protect it, after first sterilizing it with bichloride of mercury. If the pulp should be found to be exposed, or nearly so, I make a medicated soothing pad, consisting of eugenol, oxide of zinc, and a few threads of Japanese paper or cotton-wool. This I place next the pulp, and, after taking up the surplus moisture from the pad with absorbing paper, fill the cavity with soft cement with as little pressure as the case will allow. I prefer Weston's for this kind of work; vaseline is good also; but a cement which does not knead up softly should not be used. I know there are many other ways of treating such cases, but this seems to me to answer all the conditions.

If a case presents itself where the tooth has given pain not persistent, and we find a prompt response to moderately cold water and yielding to a soothing application, my plan is to treat in the same way as for the other class, hoping for the best. But so much depends on circumstances and the individual case, the constitution and health of the patient, the location of the cavity, whether accessible or not. In this class we must use our judgment and be careful. If the patient has a violent and jumping toothache, aching persistently, especially at night in bed, I think the proper thing to do is to devitalize the pulp. I first apply something to reduce the pain, such as carbolic acid and morphine, oil of cloves, eugenol, or Rosenthal's remedy. I prefer eugenol, as it seems to leave the pulp in a receptive state for the arsenic. The latter I prefer to other agents, because it leaves the dead pulp in a better state for removal. At times neuralgia is more or less associated with trouble arising from inflammation of the pulp, which, however, usually disappears after direct treatment to the tooth.

Often when a case is presented, it is difficult at first sight to know if it is really a case of inflammation of the pulp, or if, it being dead, inflammation of the alveolo-dental membrane has commenced. The patient is feverish and apprehensive, and incapable of giving much assistance in the diagnosis. If it is a case of live or dead pulp, my plan is, that where there is a cavity of greater or less depth, to test with cold water, watching the expression of the patient; if the pulp is alive there is generally no mistaking the fact, and that point is settled; but in those cases where the pulp is affected by an improper filling which has developed trouble, we may be obliged to arrive at the truth by the process of exclusion. I think it is best to decide that where there is no marked indication there is inflammation of the alveolo-dental membrane. We must search for cavities, sound suspected teeth to develop tenderness if any exists. We must study the color of the gum, must interrogate the patient as to the symptoms, and when we find as a result of the examination that a certain tooth is more sensitive than others to concussion, and that the patient complains of a dull, heavy pain, and reports that the tooth seems to be longer than the others, and that drinking of hot liquids was painful, and cold not so, we may, I think, with confidence bore into that tooth to give a vent to the confined gas, thus giving relief to the patient. If the vent gives no relief by removing the pressure, we must "do all things to conciliate; failing in that, all things to crush." Toothache of this kind is one of the most distressing ills that human flesh is heir to, and owing to its situation and the nature of it, it is often difficult to give that speedy relief the patient longs for. Owing to the pain, and the absorption into the system of more or less poison, constitutional disturbance of a greater or less degree is associated with this kind of trouble, and must be taken into consideration.

The first thing indicated is to reduce the inflammation and prevent its further progress. If suppuration has not already advanced too far, blistering pads (I make mine of red pepper and cocaine, sewed up in little bags nearly covered on the surface with chlora-percha, or strips of felt covered with wet gum tragacanth and pepper dusted over that; the gum softens in water, but does not dissolve), aconite and iodine, or strong iodine, can also be applied. I recommend a hot foot-bath, a dose of Epsom salts, and six-grain doses of antipyrin, or apply one or more leeches over the root. A lotion of cocaine, aconite, chloroform, and iodine, rubbed somewhat forcibly on the gum with the end of the finger, has a benumbing effect. Failing to get relief within a reasonable time by our efforts to reduce the inflammation, we must now turn our attention to hastening the process of suppuration and the discharge of the pus. Hot poultices,[1] having two or three drops of laudanum on each, should be applied, so as to assist the pointing to the right direction. If the pain is persistent in spite of these efforts, it is well to give an opiate. I recommend to be taken before going to bed fifteen or twenty drops of laudanum, or chloral hydrate ten grains, and bromide of potassium ten grains. Usually after the sleep thus produced the patient has no more pain. A subcutaneous injection

---

[1] We presume the writer means upon the gum. Poultices upon the cheek are not admissible.—ED.

of morphine (one-sixteenth of a grain) would answer the same purpose, and sometimes better.

I had a case in point to illustrate its efficacy. A lady sent for me who was suffering greatly on account of an upper wisdom tooth on which had been placed a Richmond crown. The pain defied every effort to reduce it until she had received two injections of morphine, after which, on awaking from her sleep, the pain had entirely left, and there was no suppuration. In these cases of crowns and bridges we have not the alternative of extraction. The question of extraction is an important one,—to know when it is expedient and proper to resort to it.

There are hopeless cases where the use of the forceps would be a mercy, and a possible insurance against much misery in the future. But judgment, qualified by honesty of purpose, should rule before the dentist yields to the suggestions of the patient and his own convenience, and extract a tooth that might, if properly treated, be retained as a useful member of the dental arch.

Erosion or wearing down of the crowns often gives rise to more or less annoyance, if not positive pain, to the patient. This condition of things, often found in the mouths of strong and healthy people, indicates a want of proper tone and solidity of the teeth, or of a solvent in the mouth which acts upon them. The only thing to do is to cover with gold. Chloride of zinc gives temporary relief, and so does the nitrate of silver. But if the case be beyond filling, the nerve should be killed and removed. The same with erosion on the buccal sides of teeth, which I consider as being different from the other class, as the erosion seems to be local and confined. The solvent in this case must come from the mucous membrane directly opposite and in contact. In cases where all of the teeth seem to be sound, but their necks exposed and the gum in an unhealthy state, it is difficult sometimes to locate the pain. The patient himself is not sure of the tooth. Sounding gives no response, cold water sets all the teeth aching, as does cold air. Ordinarily, the pain passes away with the effects of the exciting cause; at others, however, it is persistent. The cause of it is exposure,—not enough covering over the cementum, which is not as thick over the nerve or as dense as dentine, and needs the further protection of bone and gum. In such cases we must be patient in the diagnosis to isolate the tooth giving the present trouble. I have given relief in such cases by boring into the tooth and inserting a little eugenol, sealing up with gutta-percha. This method has the advantage also of testing the tooth as to its vitality. In some cases, however, where the destruction of tissue is very great, the only thing to do is to kill the pulp, after being sure of the right tooth. In all cases of this kind, and in those where sympathy might tend to mislead one, there is nothing for it but the exercise of patience and perseverance. I had recently some experience myself in regard to being misled by sympathy. I had a toothache which I thought was in the left superior canine. Paradoxical as it might seem, I rejoiced at the idea. I wanted to experience clinically on myself the different phases and symptoms of toothache. I did not ask for sympathy, which is not usually given to dentists, but when I was unable to find any cause for the pain myself, and it was growing unbearable, I consulted my friend, Dr. Schaffner, who, not finding anything apparently the matter with that tooth, commenced sounding all the teeth on that side, and when he reached the wisdom tooth I responded. Up to that time I had no suspicion that there was anything wrong with that tooth.

We must in these cases, which include persons of the rheumatic and gouty diatheses, women in gestation, etc., proceed with caution, and in cases of doubt do too little rather than risk doing too much; be governed by general principles, and for the moment treat the parts topically, and recommend systemic treatment. We are a long way off as yet from perfect knowledge; we must leave some work for the dental societies and the investigators of the future. I would much rejoice, in the interest of suffering humanity, if we could discount the future a little, and obtain a way by which the agonized patient, in a case of acute periodontitis, for example, could be surely, immediately, and painlessly relieved.

